# Age-Specific Lipid and Fatty Acid Profiles of Atlantic Salmon Juveniles in the Varzuga River

**DOI:** 10.3390/ijms17071050

**Published:** 2016-06-30

**Authors:** Svetlana A. Murzina, Zinaida A. Nefedova, Svetlana N. Pekkoeva, Alexey E. Veselov, Denis A. Efremov, Nina N. Nemova

**Affiliations:** Institute of Biology, Karelian Research Centre of the Russian Academy of Sciences, Petrozavodsk 185910, Russia; znefed@krc.karelia.ru (Z.A.N.); pek-svetlana@mail.ru (S.N.P.); veselov@krc.karelia.ru (A.E.V.); denisefremov@list.ru (D.A.E.); nemova@krc.karelia.ru (N.N.N.)

**Keywords:** lipids, fatty acids, ontogenesis, Atlantic salmon, the Varzuga River, Arctic Rivers

## Abstract

The age-specific lipid and fatty acid profiles of juvenile Atlantic salmon at different ages (0+, 1+, and 2+ years) after hatching from nests located in the mainstream of a large Arctic River, the Varzuga River, and resettling to the favorable Sobachji shoal in autumn before overwinter are herein presented. The contemporary methods of the lipid analysis were used: thin layer chromatography and gas chromatography. The results show that the stability of the regulation of important functions in developing organisms is maintained through structural alterations in lipids. These alterations can be considered as a sequence of the modifications and changes in the ratios of certain lipid classes and fatty acids constituents. In general, changes in the lipids and fatty acids (FAs) maintained the physiological limits and controls through the adaptive systems of the organism. The mechanisms of juvenile fish biochemical adaptation to the environmental conditions in the studied biotope include the modification of the energy metabolism and anabolism, and here belongs to the energy characteristics of metabolic processes.

## 1. Introduction

Lipid status is a biochemical indicator allowing the characterization of early ontogenesis success in fishes and of processes involved in the heterogeneity of fish [1]. Fatty acids (FAs) are components of lipids that mobilize and rapidly involve the adaptive and preadaptive reactions of an organism to changes in the environment [2]. In fish, which evolutionarily adapt to low temperatures, high levels of polyenoic fatty acids (PUFAs) are genetically predetermined to maintain the optimal metabolism and functioning of ectotermic organisms to life at high latitudes [3,4]. Polyunsaturated fatty acids are important for organisms for two main reasons. Firstly, they modify the physical structure and regulate the physical condition (microviscosity) of the biomembrane; therefore, fatty acids are part of acclimation or adaptation processes occurring in response to changes in the environment. Secondly, oxidized derivatives regulate many physiological processes in cell and tissues [5,6].

The fatty acids of living organisms from low trophic levels are determining the fatty acid profile of lipids in fish [7]. Aquatic invertebrates are the main food, generating essential PUFAs, such as linoleic, oleic, arachidonic, eicosapentaenoic, and docosahexaenoic acid [8,9]. A considerable number of freshwater fish, including Salmonidae, have elongation and desaturation enzymes to convert 18:3ω-3 into 20:5ω-3 and 22:6ω-3, and 18:2ω-6 into 20:4ω-6 [8,9]. In fresh water, Atlantic salmon feed on different invertebrates that are rich in 18:2ω-6 and 18:3ω-3 but have low levels of 20:5ω-3 and 22:6ω-3. After salmon migration to the sea, other marine invertebrates that are rich in 20:5ω-3 and 22:6ω-3 become the primary food ration of these fish [8]. Increased amounts of 20:4ω-6, 20:5ω-3, and 22:6ω-3 fatty acids were detected in functionally important salmon organs and tissues. The results of imitation modeling, the Monte Carlo method, suggested that 20:4ω-6, 20:5ω-3, and 22:6ω-3 fatty acids play important roles in maintaining the optimal surrounding for membrane-bound enzymes [10].

One of the commercially important species among Salmonids is the Atlantic salmon, which inhabits riverine ecosystems at high latitudes. The ontogenesis of salmon occurs in the rivers, characterized by considerable morphological and physiological differentiation and significant changes in metabolism. The Atlantic salmon has a complex life cycle: adults experience a fattening period in the sea and subsequently migrate to large rivers (such as the Varzuga River) and numerous tributaries to spawn. Such species of salmonids are considered specific integrators of marine and freshwater ecosystems: anadromous Pacific salmon accumulate and transport many general and essential elements to freshwater ecosystems, thereby maintaining many other organisms [11]. The rivers of the Kola Peninsula are one of the places in the world with wild forms and large populations of Atlantic salmon (*Salmo salar* L.). Studies of the lipid and fatty acid profiles of these fish are important to understand the biochemical mechanisms of homeostasis maintenance in juvenile salmon during postembryonic development in nature. Moreover, the results of these studies might be used to monitor the resettlement of young salmon among microbiotopes in the river, distinguished by ecological and hydrologic conditions.

The aim of the present study was to present the age-specific lipid and fatty acid profiles of juvenile Atlantic salmon at different ages (0+, 1+, and 2+ years) after hatching from nests located in the mainstream of a large Arctic river, the Varzuga River, and resettling to the favorable Sobachji shoal in autumn before winter. Notably, we investigated the biochemical status of Salmonids living in natural waters, where these organisms are exposed to a combination of ecological, trophic, and hydrological factors.

The differentiation of Salmonids in terms of biochemical status is one of the key factors for their active growth, development, and adaptive potential, securing the life and survival of this species. The investigated biochemical parameters can be further used to study the early development mechanisms in Salmonids to assess the growth rates and physiological statuses of these fish at different stages of the life cycle.

## 2. Results

The parameters of lipid metabolism, such as the total lipids (TLs), storage lipids—triacylglycerols (TAGs), and cholesterol esters (EfCHOLs), membrane lipids—phospholipids (PLs) and cholesterol (CHOL), and the fatty acids (FAs) of total lipids were examined in Atlantic salmon juveniles of different ages (0+, 1+, and 2+ years).

### 2.1. Total Lipids and Certain Lipids Classes

In October, the TL level ranged from 19.6%–16.7% dry weight with dominating stored TAGs (8.8%–9.9% dry weight) and membrane PLs (4.9%–7.0% dry weight) in salmon juveniles of different ages (0+, 1+, and 2+ years) from the Sobachji shoal (Table 1). Herewith, higher levels of TLs (reflecting TAGs and PLs) were determined in fingerlings (0+). Fish at 1+ and 2+ years had higher levels of cholesterol esters compared with younger fish (0+). Certain lipid ratios were age-specific and increased in fish aged 0+ to 2+ years: CHOL/PLs ranged from 0.3–0.4, TAGs/PLs ranged from 1.4–1.9, and TAGs + EfCHOLs/PLs + CHOL ranged from 1.2–1.4 (Table 1). The fingerlings had higher ratio of PLs/TAGs and lower ratio of CHOL/PLs and TAGs + EfCHOLs/PLs + CHOL in comparison to fish at 1+ and 2+ ages.

Thus, differences in the levels of TLs and certain lipid classes and length–weight characteristics were observed among young salmon of different ages especially in fingerlings.

### 2.2. Fatty Acids of Total Lipids

We detected more than 40 fatty acids, including minor molecules, in a fatty acid spectrum of total lipids of young Atlantic salmon of different ages inhabiting the Sobachji shoal. PUFAs dominated, ranging from 33.9% to 40.3% in the fatty acid spectrum of the studied fish. Fatty acids of the ω-3 family were prevalent among PUFAs, showing 24.0%–29.7% of the total FAs, while lower amounts were detected in fingerlings (0+) and higher amounts were detected in parr (1+ and 2+) (Table 2).

Three dominant PUFAs were observed: 18:3ω-3 (7.6%–9.1%), 20:5ω-3 (4.4%–6.2%), and 22:6ω-3 (5.4%–8.6%). The increased amount of essential 20:5ω-3 and 22:6ω-3 PUFAs correlated with decreased levels of the metabolic precursor 18:3ω-3, thereby increasing the 22:6ω-3/18:3ω-3 ratio in young fish.

The level of ω-6 PUFAs in which 18:2ω-6 and 20:4ω-6 were major species vary in fish of different ages (Table 2). A significant increase in 20:4ω-6 and a decrease in 18:2ω-6 was observed in fish at age 1+. Herewith, the variation in the 20:4ω-6/18:2ω-6 ratio, indicating the conversion of 18:2ω-6 to 20:4ω-6 in young salmon, increased in fish aged 1+ and decreased in fish aged 2+.

In general, the level of ω-6 PUFAs was 9.3%–9.9% of the total FA and did not vary much during growth and development, while the level of ω-3 FAs increased with age (from 24.0% to 29.7%). Thus, the ω-3/ω-6 PUFA ratio significantly increased in fish at age 1+.

In addition to PUFAs in the total lipids of young salmon, the monoenic fatty acids (MUFAs) level was high, ranging from 30.4% to 32.3% of the total FA; among MUFAs, 16:1ω-7, 18:1ω-7, and 18:1ω-9 FAs were dominating (9.1%–10.1%, 7.1%–7.4%, and 11.8%–13.2%, respectively) (Table 2). Significant variations in the amount of MUFAs (primarily, 18:1ω-9) were observed in the studied fish of different ages: the MUFAs decreased in parr (1+) and increased in fish at 2+.

The saturated fatty acids (SFAs) ranged from 28.9%–33.2% of the total FAs, in which 16:0 and 18:0 were dominant (Table 2). An increased level of these FAs, and certain minor FAs (17:0, 20:0), was observed in the salmon fingerlings, and these levels decreased in fish aged 1+, and 18:0 FAs decreased in young salmon aged 2+.

## 3. Discussion

### 3.1. Total Lipids and Lipid Classes

Comparing the level of TLs and their certain lipid classes in young Atlantic salmon inhabiting the Sobachji shoal of Varzuga River showed that the fingerlings had a higher lipid status in which the PLs and TAGs concentrations were high. Previous analogous studies in October 2009 examining the lipid status of fingerlings after hatching showed active resettling in another biotope, the Arenga shoal of the Varzuga River [12,13,14]. We compared the lipid status of the fingerlings from the Sobachji and Arenga shoals and observed that the fingerlings inhabiting the Arenga shoal had lower TL levels, reflecting low TAGs and EfCHOLs storage and lower length–weight characteristics. These differences might reflect the phenotypic reaction of fish to specific ecological (primarily, biotic factors, such as the quality, quantity, and availability of food) and hydrological conditions of certain biotopes, which contribute to the adaptation of young fish to the environment. Presumably, the metabolic heterogeneity, which appears in salmonids at the earliest developmental stages in connection with their dispersal and foraging patterns and a number of other ecological factors, results in the divergence of individuals by the energy metabolism levels and supports further growth and development in certain environments.

During the growth and development of fish at different ages, the TAGs/PLs and TAGs + EfCHOLs/PLs + CHOL ratios, indicating the intensity of storage lipid metabolism, increased, showing the increased energetic abilities (potential) of the developing fish. The primary depot of storage lipids (TAGs) in Salmonids is muscle, and the amount of lipids in these tissues reflects the physiological condition of the organism, including fish mobility [3,15]. These lipids determine the ability of these organisms to catch and effectively digest food to maintain the further growth and development of young fish [5,16]. In this study, underwater observations during sampling showed that the intensity of feeding of the juvenile fish (the number of food shots within 15 min) was stable for each age group of fishes. It was shown that foraging conditions are more favorable for young age groups (0+) of salmon in small tributaries of the Varzuga River comparing to the mainstream [17]. The quantitative level of TAGs in fish, including fatty Salmonids, shows food supply and energetic reserve accumulation, which are important for further overwintering in the Arctic Rivers, such as the Varzuga River [13,14,18], while the qualitative profile of TAGs is tightly associated with the species composition of food objects. These storage lipids also play important roles in the adaptive reactions of organisms associated with membrane lipid modifications [1]. The salmon juveniles at different ages inhabiting one biotope (the Sobachji shoal) showed no distinctions in the level of TAGs in their bodies, reflecting the adequate food supply and effective food ingestion by these fish. Thus, the biotic conditions in this biotope are favorable for these organisms. Significant variations in the amount of another form of storage lipid, EfCHOLs, (decreased in fish at age 1+ and increased in fish at age 2+) might be associated with some differences in the food species composition that derive these lipid classes in young fish. Indeed, natural food objects have a significant amount of EfCHOLs and necessary FAs compared with artificial nutrition [19].

Importantly, we assessed the physiological role of lipids and certain lipid classes, the qualitative ratios of which are critical and significant, to show the specific microviscosity of the membrane and variations in physiological limits at different stages of development. The CHOL/PLs ratio increased in young fish at ages 0+, 1+, and 2+ (0.3; 0.4; 0.4, respectively), reflecting decreased levels of PLs and stable amounts of CHOL in autumn. We also assume that this observation also reflected the activation of the biochemical mechanism maintaining the necessary functional activity of membrane-bound enzymes in response to the environment (temperature) and physiological changes in organism during growth. In the present study, the decreased TLs observed in young salmon aged 1+ and 2+ in October was also previously detected in the juveniles of *Parasalmo mykiss* and *Oncorhynchus kisutch* from the Northwest of Kamchatka in autumn [18].

The decreasing amount of TLs (primarily, PLs), and the increased TAGs/PLs and CHOL/PLs ratios in parr aged 1+ and 2+ reflected the physiological change during growth and development, furthered the parr-smolt transition, and partly reflected the quality of the freshwater growing area of the Sobachji shoal in this season. Variations in the levels of certain lipids among fishes of different ages and in one age group might show the differences in the rates of certain biochemical reactions involving the metabolism and modification of lipids and fatty acid constituents associated with phenotypic diversity.

### 3.2. Fatty Acids of the Total Lipids

The fatty acid profile is important for the growth and development of juvenile fish and is primarily determined based on the fatty acids of food and the ability of fish to undergo modifications depending on a specific environment. PUFAs were dominating in salmon juveniles, showing the physiological importance of these molecules in this organism [2]. The increased levels of essential 20:5ω-3 and 22:6ω-3 in the fish examined in the present study were correlated with decreased levels of the metabolic precursor 18:3ω-3. Freshwater fish, compared with marine food-derived 18:3ω-3, more actively convert this molecule to long-chain PUFAs [2,20]. The 22:6ω-3/18:3ω-3 ratio, indicating the level of ω-3 PUFA metabolism, increased from 0.6 to 1.1 with age in the studied fish, suggesting that the increased amount of long-chain PUFAs in the juveniles at age 1+ might be associated with the start of fatty acid profile modification from a freshwater type to a marine type, consistent with the results of a previous study in fish aged 2+ and smolts (3+), inhabiting another biotopes of the Varzuga River [12,13,14]. Changes in the FA spectrum reflecting increasing PUFAs promote smoltification and the further migration of smolts to the sea [21]. The long-chain PUFAs, 20:5*n*-3 and 22:6*n*-3, determine the switch of the FAs of total lipids to marine types [2,3].

A comparison of the concentration of the 20:4ω-6 FA (ranging from 2.13% to 2.93% of total FAs) and the metabolic precursor 18:2ω-6 (ranging from 5.12% to 5.52%) in juvenile salmon of different ages showed the low activity (or complete absence) of linoleoyl-CoA-desaturase, which plays a significant role in the pathway of 18:2ω-6 to 20:4ω-6 [22]. A previous study [12] showed that the metabolism of PUFAs containing 18 C atoms in their structure to long-chain PUFAs is difficult in Salmonids, and, in the present study, we detected low levels of ω-6 PUFAs (primarily 20:4ω-6) compared with the higher levels of the precursor 18:2ω-6 in the studied fishes. However, the 20:4ω-6/18:2ω-6 ratio, indicating the intensity of the metabolic conversion of ω-6 PUFAs, showed the increased trend in the studied fish at ages 0+, 1+, and 2+ (0.4%, 0.6%, and 0.5%, respectively). The effectiveness of this biosynthesis depends on the concentration of 18:2ω-6 and the activity of desaturases and elongases [20,23,24]. Arachidonic FA is a precursor of physiologically active endohormones (prostaglandins, thromboxanes, and leukotrienes), regulators of different processes in organisms, including reproduction, stress responses, and growth [5,25,26]. Considering the role of 20:4ω-6 in the synthesis of the important endohormones, an increasing 20:4ω-6/18:2ω-6 ratio indicates increasing synthesis, particularly in the juvenile salmon aged 1+.

Increasing the ω-3/ω-6 PUFAs in juveniles at age 1+ showed changes in the functional conditions of the biomembrane of tissues during the growth and development of the fish. Importantly, the optimization of ω-3/ω-6 PUFAs is significant due to competitor relations in metabolism. The specific role in the regulation of the direction of the metabolic pathways is the concentration and the ratio of the saturated and unsaturated FA, primarily PUFAs, in the organism, which further determines the microviscosity of the biomembrane and maintains the optimal micro surrounding for the activities of the membrane-bound enzymes. The SFA/PUFA ratio decreased in salmon juveniles (from 0.98—fish at age 1+ to 0.78—fish at age 2+), reflecting increased levels of ω-3 PUFAs. We detected the same dynamic in a previous study on salmon juveniles (0 +, 1+, 2+, and 3+ smolts) from other biotopes of the Varzuga River (the Arenga tributary and the littoral of the Varzuga River) [14].

## 4. Materials and Methods

The Sobachji shoal is located 24.6 km away from the mouth of the river (picture). The extension of the shoal is approximately 600 m, and the width ranges from 120–160 m. The shoal was low and deep, and the depth ranges from 0.20 to 1.30 m, averaging 0.35–0.65 m. The current varies from 0.50–1.2 m/s. The Sohachji shoal is a Salmonid spawning location inhabited by young fish of different ages: 0+, 1+, 2+, and 3+ years. Dwarf salmon also live here (4+ and 5+ years). The density of young fish varies from 22–54 individuals/100 m^2^ (0.7 individuals/m^2^). In autumn, the number of drift organisms decreases, and the parr initially feed on benthic organisms and the larvae of Trichoptera, the predominant molluscs observed in autumn. The Varzuga River is at the level of the polar circle. Spawning of salmon in this river occurs in the II–III decade of September when water temperature decreases from 7.0–8.0 °C to 3.0 °C. In the I decade of October, the water temperature decreases to 2.0–1.5 °C and parr of salmon disappear under boulders and stay inactive as during the winter.

Juveniles of salmon at different ages (0+, 1+, 2+ years) were collected in autumn (October 2014) in the Sobachji shoal of the mainstream of Varzuga River. An electrofishing device (Zippin, Fa-2, Norway) was used to collect fish. To avoid the effect of electrofishing, fingerlings were held for 24 h in cages located in the mainstream portion of the river (numbers of fish are indicated in Table 1). The age of fish was determined by scales. The annual monitoring of the density of the distribution of different age groups of salmon parr was carried out in the Varzuga River. Fish and tissue samples were homogenized individually in 10 volumes (10 mL each) of 96% ethyl alcohol mixed with 0.001% of antioxidant (ionol). The volume of solvent was 10 times the weight of a sampled fish. Sample homogenates were placed in glass vials and stored at 4 °C until delivery to the laboratory.

The material was then fixed in a solvent of chloroform:methanol (2:1, *v*/*v*), and the total lipids (TLs) were extracted using the method of Folch et al. [27]. The residues recovered after lipid extraction from the tissues were dried over phosphoric anhydride until the samples reached a constant weight. The residues were weighed (*X*1) to determine the approximate percentage of total lipid on a dry-weight basis:

Total lipids (% dry weight)=X2×100/(X1+X2)

where *X*1 = residue weight (g), and *X*2 = lipid extracted (g).

The lipid status of each fish was evaluated by determining the content of total lipids, triacylglycerides (TAGs), phospholipids (PLs), cholesterol (CHOL), cholesterol esters (EfCHOLs) and the fatty acid (FA) spectrum of the total lipids.

Thin-layer chromatography was used to identify the lipid classes: PLs, TAGs, CHOL, and EfCHOLs. After drying, the chromatogram was developed in iodine vapor, which stains lipids yellow. These molecules were quantified using a modified hydroxamate method [28], which involves the formation of dark-brown complexes of trivalent iron ions with hydroxamic acid through ester-bonding between the lipids and hydroxylamine [29]. The stain intensity was measured using a spectrophotometer (SF-2000, OKB Spectr, St. Petersburg, Russia) at a wavelength of 540 nm. The quantitative determination of CHOL was determined according to the method described in [30] using trichloroacetic iron dissolved in perchloric acid. The stain intensity was measured using a spectrophotometer at a wavelength of 550 nm. Standards (Sigma Aldrich, St. Loius, MO, USA) for thin-layer chromatography were used to distinguish the lipid classes on the plates.

The fatty acid composition of the total lipid extracts was analyzed by gas-liquid chromatography. Fatty acid methyl esters (FAMEs) were identified using a “Chromatec-Crystall-5000.1” (Chromatec, Yoshkar-Ola, Russia) gas chromatograph with a flame-ionization detector and a Zebron ZB-FFAP capillary gas chromatographic column (Phenomenex, Torrance, CA, USA). An isothermal column configuration was used; the temperatures of the detector and evaporator were 240 and 240 °C, respectively. The internal standard was 22:0 FA. Chromatek-Analytik-5000.1 software (Chromatec) was used for recording and integrating the data. FAMEs were identified with standard mixtures Supelco^®^ 37 Component FAME mix (Sigma Aldrich, St. Loius, MO, USA) and by comparing the equivalent lengths of carbon chains and table constants according to Jamieson [31].

The research was carried out using the facilities of the Equipment Sharing Centre of the Institute of Biology, KarRC of RAS, Petrozavodsk, Russia. The results are given as the average ± standard deviation (SD). The data were analyzed to determine whether they exhibited a normal distribution. The differences between the means of the lipid and fatty acid parameters of the fish of three age classes were tested by ANOVA (*p* ≤ 0.05).

## 5. Conclusions

The maintenance of the living functions of juvenile salmon inhabiting one biotope during growth and development in autumn is provided by the modifications of the ratios of lipid classes and fatty acid constituents. A decreased level of TLs due to PLs, variations in EfCHOLs, and increased ratios (CHOL/PLs, TAGs/PL, and TAGs + EfCHOLs/PLs + CHOL) were observed in the juvenile salmon. These results showed the different rates and intensities of metabolic processes during growth and development in fishes in autumn. Individuals (fingerlings) with the initially higher level of the energy metabolism are more active in foraging and growing, and sooner reach a certain size and metabolic status, when they are ready to smoltify.

The fingerlings of Atlantic salmon compared with the parr (1+, 2+) showed an increased level of TLs (primarily, PLs and TAGs) resulting from the PL/TAG ratio, and the sum of SFAs (primarily, 16:0 and 18:0) increased. These findings reflected the active processes of the biosynthesis of membrane PLs and fatty acid constituents at concrete age periods, associated with a high ratio of 16:0/18:1ω-9, indicating the intensity of lipid metabolism.

Increased amounts of the long-chain PUFAs, 20:5ω-3, 22:5ω-3, and 22:6ω-3, and the 22:6ω-3/18:3ω-3 and ω-3/ω-6 ratios in parr aged 1+ indicate the main biochemical aspect showing the start of the FA spectrum modification from the freshwater type to the marine type. We previously observed the same switch but in older fish (age 2+ and 3+) inhabiting another biotope (the Arenga shoal of the Varzuga River).

The results obtained in the present study shows that the stability of the regulation of important functions in developing organisms is maintained through structural alterations in lipid systems. These alterations can be considered as a sequence of the modifications and changes in the ratios of certain lipid classes and fatty acids constituents. In general, changes in the lipids and FAs maintained the physiological limits and controls through the adaptive systems of the organism.

Thus, the results of the present study complement the previously obtained data on the effect of the environment (“niches”) on the growth and development of juvenile Salmonids. The mechanisms of juvenile fish biochemical adaptation to the environmental conditions in the studied biotope include the modification of the energy metabolism and anabolism, and the key role here belongs to the energy characteristics of metabolic processes. Presumably, the metabolic heterogeneity observed in Salmonids at the earliest developmental stages associated with the dispersal and foraging patterns of these organisms and a number of other ecological factors resulted in the divergence of individuals through energy metabolism levels.

## Figures and Tables

**Table 1:** Total lipids and lipid classes (% dry weight) of juvenile Atlantic salmon at different ages (0+, 1+, and 2+ years) in the Sobachji shoal (the Varzuga River). Data are presented as average ± SD (standard deviation).

Parameter/Age of Fish, Years	0+	1+	2+
Number of fish	15	14	15
Length, sm	0.3 ± 0.0	9.1 ± 0.2 ^A^	11.3 ± 0.2 ^B,^^С^
Weight, g	0.2 ± 0.0	5.6 ± 0.4 ^A^	11.0 ± 0.4 ^B,^^С^
TLs	19.6 ± 0.7	17.0 ± 0.5 ^A^	16.7 ± 1.0 ^B^
PLs	7.0 ± 0.6	5.0 ± 0.2 ^A^	4.9 ± 0.3 ^B^
TAGs	9.9 ± 0.9	9.6 ± 0.7	8.8 ± 1.0
EfCHOLs	0.7 ± 0.1	0.4 ± 0.1 ^A^	1.0 ± 0.1 ^B^
CHOL	2.0 ± 0.2	2.0 ± 0.1	2.1 ± 0.1
PLs/TAGs	1.0 ± 0.2	0.6 ± 0.1 ^A^	0.6 ± 0.1 ^B^
TAGs/PLs	1.4 ± 0.2	1.9 ± 0.2 ^A^	1.8 ± 0.2 ^B^
CHOL/PLs	0.3 ± 0.1	0.4 ± 0.1 ^A^	0.4 ± 0.1 ^B^
TAGs + EfCHOLs/PLs + CHOL	1.2 ± 0.1	1.4 ± 0.1 ^A^	1.4 ± 0.1 ^B^

^A^ Indicates significant differences (*p* ≤ 0.05) between fish at 0+ and 1+*;* ^В^ Indicates significant differences (*p* ≤ 0.05) between fish at 0+ and 2+; ^С^ Indicates significant differences (*p* ≤ 0.05) between fish at 1+ and 2+; TLs: Total lipids; PL: Phospholipids; TAGs: Triacylglycerols; EfCHOLs: Cholesterol esters; CHOL: Cholesterol.

**Table 2:** Fatty acid spectrum (% total FAs) of juvenile Atlantic salmon at different ages (0+, 1+, and 2+ years) in the Sobachji shoal (the Varzuga River). Data are presented as average ± SD.

Age of Fish, Years/Parameter	0+	1+	2+
Number of fish	15	14	15
14:0	2.1 ± 0.1	1.9 ± 0.1	2.1 ± 0.2
16:0	19.8 ± 0.7	17.3 ± 0.2 ^A^	18.0 ± 0.6
17:0	1.1 ± 0.0	0.9 ± 0.0 ^A^	0.8 ± 0.1 ^B^
18:0	8.3 ± 0.3	7.2 ± 0.1 ^А^	6.9 ± 0.2 ^В^
20:0	1.3 ± 0.1	1.2 ± 0.0	1.2 ± 0.0
∑SFA	33.2 ± 1.2	28.9 ± 0.1 ^А^	29.4 ± 0.9 ^В^
16:1ω-9	0.7 ± 0.0	0.6 ± 0.0 ^А^	0.6 ± 0.0 ^В^
16:1ω-7	9.6 ± 0.4	9.1 ± 0.3	10.1 ± 0.5
18:1ω-9	13.2 ± 0.4	11.8 ± 0.2 ^А^	12.3 ± 0.5
18:1ω-7	7.3 ± 0.2	7.1 ± 0.1	7.4 ± 0.3
20:1ω-9	0.4 ± 0.0	0.4 ± 0.0	0.5 ± 0.1
20:1ω-7	0.2 ± 0.0	0.3 ± 0.0 ^А^	0.2 ± 0.0 ^В,C^
∑MUFA	32.3 ± 0.8	30.4 ± 0.3 ^А^	32.1 ± 0.5 ^С^
18:2ω-6	5.5 ± 0.1	5.1 ± 0.1 ^А^	5.1 ± 0.3
20:4ω-6	2.1 ± 0.2	2.9 ± 0.1 ^А^	2.5 ± 0.2 ^В,C^
∑ω-6 PUFA	9.4 ± 0.3	9.9 ± 0.2	9.3 ± 0.0 ^С^
16:2ω-3	0.5 ± 0.0	0.6 ± 0.0 ^А^	0.7 ± 0.1 ^В^
18:3ω-3	9.1 ± 0.5	8.5 ± 0.2 ^A^	7.6 ± 0.4 ^В,С^
18:4ω-3	1.0 ± 0.1	1.4 ± 0.0 ^А^	1.4 ± 0.1 ^В^
20:5ω-3	4.4 ± 0.4	6.2 ± 0.1 ^А^	6.2 ± 0.5 ^В,С^
22:6ω-3	5.4 ± 0.6	8.6 ± 0.4 ^А^	7.9 ± 1.1 ^В,С^
∑ω-3 PUFA	24.0 ± 1.7	29.7 ± 0.4 ^А^	28.0 ± 1.3 ^В^
∑PUFA	33.9 ± 2.0	40.3 ± 0.4 ^А^	38.0 ± 1.3 ^C^
ω-3/ ω-6 PUFA	2.5 ± 0.1	3.0 ± 0.1 ^А^	3.0 ± 0.2 ^В^
16:0/18:1(ω-9)	1.5 ± 0.0	1.5 ± 0.0	1.5 ± 0.1

^A^ Indicates significant differences (*p* ≤ 0.05) between fish at 0+ and 1+; ^В^ Indicates significant differences (*p* ≤ 0.05) between fish at 0+ and 2+; ^С^ Indicates significant differences (*p* ≤ 0.05) between fish at 1+ and 2+; SFAs: Saturated fatty acids; MUFAs: Monoenic fatty acids; PUFAs: Polyenoic fatty acids.

## Abbreviations

TLs: Total lipids
TAGs: Triacylglycerols
EfCHOLs: Cholesterol esters
PLs: Phospholipids
CHOL: Cholesterol
FAs: Fatty acids
PUFAs: Polyunsaturated fatty acids
MUFAs: Monounsaturated fatty acids
SFAs: Saturated fatty acids
